# Hindfoot motion according to subtalar compensation and ankle osteoarthritis stage analyzed by a multi-segment foot model

**DOI:** 10.1186/s13018-024-04615-0

**Published:** 2024-03-26

**Authors:** Ho Won Kang, Dae-Yoo Kim, Jung Min Kim, Gil Young Park, Dong-Oh Lee, Dong Yeon Lee

**Affiliations:** 1 Department of Orthopedic Surgery, Ewha Womans University Mokdong Hospital, Seoul, Republic of Korea; 2 Department of Orthopedic Surgery, Inje University Busan Paik Hospital, Busan, Republic of Korea; 3 Department of Orthopedic Surgery, Seoul National University Hospital, Seoul, Republic of Korea; 4 Department of Orthopedic Surgery, SNU Seoul Hospital, Seoul, Republic of Korea; 5 Department of Orthopedic Surgery, Seoul National University College of Medicine, 101 Daehak-no, Jongno-gu, Seoul, 03080 Republic of Korea

**Keywords:** Ankle, Gait analysis, Hindfoot alignment, Osteoarthritis, Subtalar compensation

## Abstract

**Background:**

The biomechanics of the hindfoot in ankle osteoarthritis (OA) are not yet fully understood. Here, we aimed to identify hindfoot motion in a gait analysis using a multi-segment foot model (MFM) according to ankle OA stage and the presence of subtalar compensation defined by hindfoot alignment.

**Methods:**

We retrospectively reviewed the medical records, plain radiographs, and gait MFM data of 54 ankles admitted to our hospital for the treatment of advanced ankle OA. Spatiotemporal gait parameters and three-dimensional motions of the hindfoot segment were analyzed according to sex, age, body mass index, Takakura classification, and the presence of subtalar compensation. Twenty ankles were categorized as compensated group, and 34 ankles as decompensated group.

**Results:**

No spatiotemporal gait parameters differed significantly according to the presence of subtalar compensation or ankle OA stage. Only normalized step width differed significantly (*P* = 0.028). Average hindfoot motion (decompensation vs. compensation) did not differ significantly between the sagittal and transverse planes. Graphing of the coronal movement of the hindfoot revealed collapsed curves in both groups that differed significantly. Compared with Takakura stages 3a, 3b, and 4, cases of more advanced stage 3b had a smaller sagittal range of motion than those of stage 3a (*P* = 0.028). Coronal movement of the hindfoot in cases of Takakura stage 3a/3b/4 showed a relatively flat pattern.

**Conclusions:**

The spatiotemporal parameters were not affected by the hindfoot alignment resulting from subtalar compensation. The sagittal range of hindfoot motion decreased in patients with advanced ankle OA. Once disrupted, the coronal movement of the subtalar joint in ankle OA did not change regardless of ankle OA stage or hindfoot compensation state.

## Introduction

Ankle osteoarthritis (OA) is a debilitating condition that generally develops after trauma [1]. Total ankle arthroplasty or ankle arthrodesis has been the standard treatment for end-stage ankle OA [2]. Meanwhile, earlier-stage ankle OA is often asymmetric, involving only part of the joint surface [3]. Therefore, many clinicians have recently attempted joint-preserving surgery, such as supramalleolar osteotomy, but the clinical outcomes were not always satisfactory [4–6].

We do not yet fully understand the biomechanics of the ankle joint, especially those with OA pathology. Talus orientation changes as ankle OA progresses. The compensatory function of the subtalar joint, which maintains the neutral alignment of the hindfoot until the intermediate OA stage, may also affect the presentation of ankle OA [7]. The biomechanics may differ according to ankle OA state, which would provide clinicians and researchers with insight regarding its better treatment.

Multi-segment foot model (MFM) analysis, which has advantages over traditional standard clinical gait analysis, is becoming popular in clinical research because the foot comprises 26 small bones [8, 9]. The additional value of MFM analysis has already been demonstrated with respect to the traditional identification of anatomical deformities in static conditions [9, 10]. Nevertheless, few studies have reported on MFM analysis in patients with advanced ankle OA [11]. We found several studies that compared gait patterns between ankle OA patients and controls [12–15]. Otherwise, no study has explored gait patterns using an MFM in ankle OA patients by severity. Therefore, here we aimed to: (1) analyze the segmental motions of the hindfoot using an MFM according to the presence of subtalar compensation from the gait of patients with symptomatic ankle OA who underwent surgery and (2) compare them by ankle OA stage. We hypothesized that the segmental motion of the hindfoot would be affected by the presence of subtalar compensation and ankle OA stage.

## Materials and methods

### Subjects

**Fig. 1:**
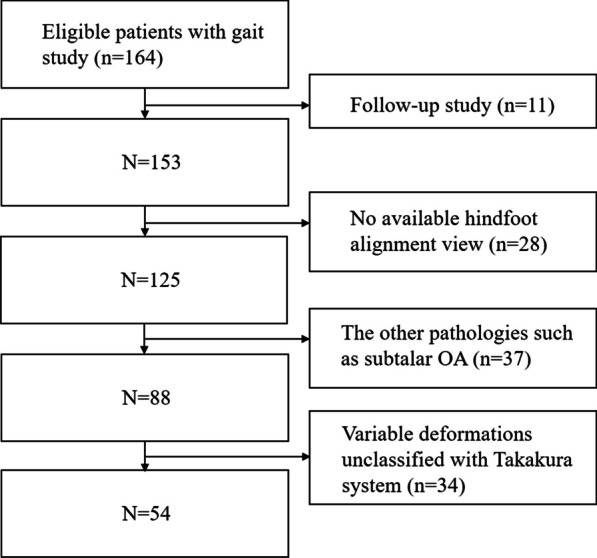
Flowchart of the selection of the study subjects among the total population for whom multi-segment foot model gait analysis data were available

All experimental protocols were approved by Seoul National University Hospital Institutional Review Board (H-1806-035-949). Owing to the retrospective nature of the study, the requirement for informed consent was waived by Seoul National University Hospital IRB. All research protocols were carried out in accordance with the Declaration of Helsinki. We reviewed the medical records, MFM gait analysis information, and radiography images of patients who were admitted to our hospital to undergo surgical treatment for advanced ankle OA. Conservative treatments such as oral medication, bracing, and injection for at least 6 months had failed in all patients at the time of admission. From 2015 to 2019, 164 ankles underwent an MFM gait study. We excluded patients with diabetes mellitus–related foot symptoms, neurological disorders, or rheumatoid arthritis as well as those who had simultaneous pathology of the other joints of the foot including the subtalar joint, midfoot, forefoot, or proximal tibiofibular joint based on radiographic imaging findings and physical examination performed by a senior orthopedic surgeon. We excluded 34 patients who had an abnormal orientation of the talus that prevented Takakura system classification including valgus OA because these OA cases would be too variable for analysis. As a result, 54 ankles (54 patients) were included in this study (Fig. 1). The mean patient age was 69.3 years; 22 of them were men. The mean body mass index was 26.3. The right side was 32 in laterality. Forty-three patients underwent total ankle arthroplasty, while 32 underwent ankle fusion. Three patients underwent supramalleolar osteotomy, and three patients underwent a modified Broström procedure, medial plication, and first metatarsal dorsiflexion osteotomy. Four patients underwent calcaneal Dwyer osteotomy, and three patients chose additional conservative treatment after admission.

### Radiographic evaluation

**Fig. 2:**
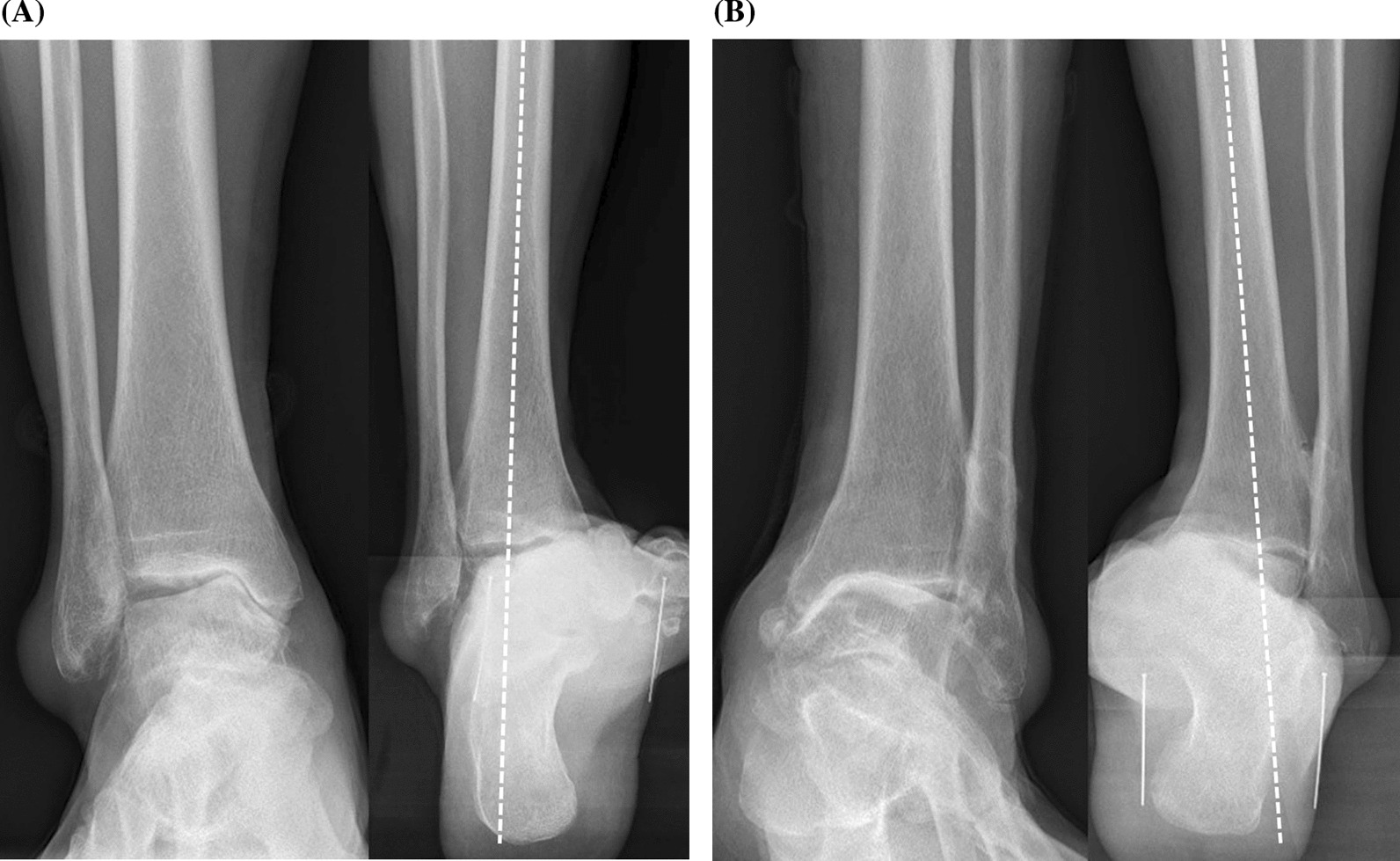
Two examples of ankle osteoarthritis stage. **A** Ankle with Takakura stage 3a and neutral hindfoot alignment caused by successful subtalar compensation. **B** Ankle with Takakura stage 3b and varus hindfoot alignment caused by subtalar decompensation

For each patient, two orthopedic surgeons with more than 5 years of experience observed the plain radiographs of the weight-bearing anteroposterior ankle and hindfoot alignment view. We employed the Takakura ankle OA classification system (Fig. 2) [16, 17]. These two surgeons finally determined the stage after discussion in the cases of discrepancy between their initial opinions. As a result, 13 patients were stage 3a, 13 were 3b, and 28 were stage 4. To determine the presence of subtalar compensation, the apparent moment arm was measured on hindfoot alignment radiography [18]. If the perpendicular distance between the longitudinal midaxis of the distal tibia and the lowest point of the calcaneus (i.e., apparent moment arm) was less than 15 mm, the ankle was classified into the compensated group; otherwise, it was classified into the decompensated group [19]. Twenty ankles were categorized as compensated group, and 34 ankles as decompensated group.

### Intersegmental angle measurements during gait using an MFM

All patients routinely underwent three-dimensional (3D) MFM analysis with 15 markers at the time of hospital admission. The instrumented 3D gait analysis with an MFM was performed according to a previously described protocol [2, 20]. Kinematic data of the foot segmental motion were monitored and obtained using Foot 3D Multi-Segment Software (Motion Analysis, Santa Rosa, CA, USA), while the patients walked at a comfortable speed along an 8-m track [2]. Three representative strides from five separate trials were used for each patient [2]. The temporal gait parameters including cadence, speed, stride length, step width, step time, and stance duration were calculated. The intersegmental positions during the eight phases of gait (initial contact, loading response, midstance, terminal stance, preswing, initial swing, midswing, and terminal swing) were collected.

### Analysis

Categorical variables such as sex and surgery type were analyzed using the chi-square test or Fisher’s exact test. All variables underwent Kolmogorov–Smirnov *Z* test analysis for normality. As a result, continuous variables including spatiotemporal gait parameters were compared between the decompensation and compensation groups using the Mann–Whitney test.

Analysis of the relationship between hindfoot segmental motion and ankle OA stage was performed using the Kruskal–Wallis test and post hoc analysis. All statistical analyses were performed using the Statistical Package for Social Science (SPSS) version 25 (IBM Corp., Armonk, NY). The level of significance was set at *P* < 0.05.

## Results

### Effect of subtalar joint compensation

Table 1 compares the demographics and spatiotemporal parameters of the study population according to the presence of subtalar compensation. Regarding the effect of subtalar compensation on gait pattern, no significant intergroup differences were noted in spatiotemporal gait parameters or demographic variables including sex and age (Table 1).

**Table 1:** Demographics and spatiotemporal gait parameters of the study population according to the presence or absence of subtalar compensation

	Decompensation (*n* = 20)	Compensation (*n* = 34)	*P* value
Sex (M:F)	13:7	15:19	0.510
Age	68.1 ± 4.4	70.0 ± 6.6	0.178
BMI^a^, kg/m^2^	26.3 ± 4.1	26.3 ± 3.5	0.816
Laterality (R:L)	14:6	18:16	0.262
Cadence, step/min	107.6 ± 8.9	105.4 ± 10.2	0.463
Speed, cm/s	80.2 ± 17.6	86.1 ± 21.9	0.197
n Speed^b^, %	51.0 ± 10.1	54.1 ± 13.0	0.185
Stride length, cm	89.5 ± 18.0	97.4 ± 20.4	0.099
n Stride length^b^, %	56.9 ± 10.1	61.2 ± 11.6	0.089
Step width, cm	13.1 ± 2.7	13.1 ± 3.3	0.844
n Step width^b^, %	8.3 ± 1.7	8.3 ± 2.0	0.929
Step time, s	0.56 ± 0.05	0.57 ± 0.06	0.463
Stance duration, %	63.2 ± 10.7	58.5 ± 17.2	0.132

^a^Body mass index

^b^n. Normalized with height

### Effect of ankle OA stage

Table 2 compares the demographics and spatiotemporal parameters of the study subjects according to Takakura ankle OA stage. Regarding the relationship between hindfoot segmental motion and ankle OA stage, the normalized step width differed significantly (*P* = 0.028). The other spatiotemporal gait parameters did not differ among the three groups, although there were significant differences in sex ratio and laterality (Table 2).

**Table 2:** Demographics and spatiotemporal gait parameters of the study population according to Takakura ankle OA stage

	Takakura stage			*P*
	3a (*n* = 13)	3b (*n* = 13)	4 (*n* = 28)	
Sex (M:F)	6:7	7:6	15:13	0.694
Age	67.5 ± 5.4	71.2 ± 7.6	69.2 ± 5.3	0.275
BMI^a^, kg/m^2^	24.8 ± 1.8	27.7 ± 4.8	26.3 ± 3.6	0.275
Laterality (R:L)	7:6	4:9	21:7	0.026^c^
Cadence, step/min	108.4 ± 11.5	109.1 ± 7.1	103.9 ± 9.6	0.137
Speed, cm/s	90.7 ± 18.7	79.3 ± 20.3	82.8 ± 21.1	0.378
n Speed^b^, %	57.3 ± 10.8	51.3 ± 12.0	51.7 ± 12.5	0.292
Stride length, cm	100.0 ± 14.4	86.4 ± 20.3	95.6 ± 21.1	0.202
n Stride length^b^, %	63.3 ± 8.2	56.0 ± 11.8	59.6 ± 11.8	0.267
Step width, cm	11.5 ± 2.5	12.8 ± 3.5	14.0 ± 2.8	0.032^d^
n Step width^b^, %	7.2 ± 1.5	8.3 ± 2.2	8.8 ± 1.7	0.028^d^
Step time, s	0.56 ± 0.07	0.55 ± 0.04	0.58 ± 0.05	0.137
Stance duration, %	64.6 ± 2.0	54.5 ± 23.0	60.9 ± 13.8	0.761

^a^Body mass index

^b^n. Normalized with height

^c^Significantly difference between stage 3b and 4

^d^Significantly differences in each groups

**Fig. 3:**
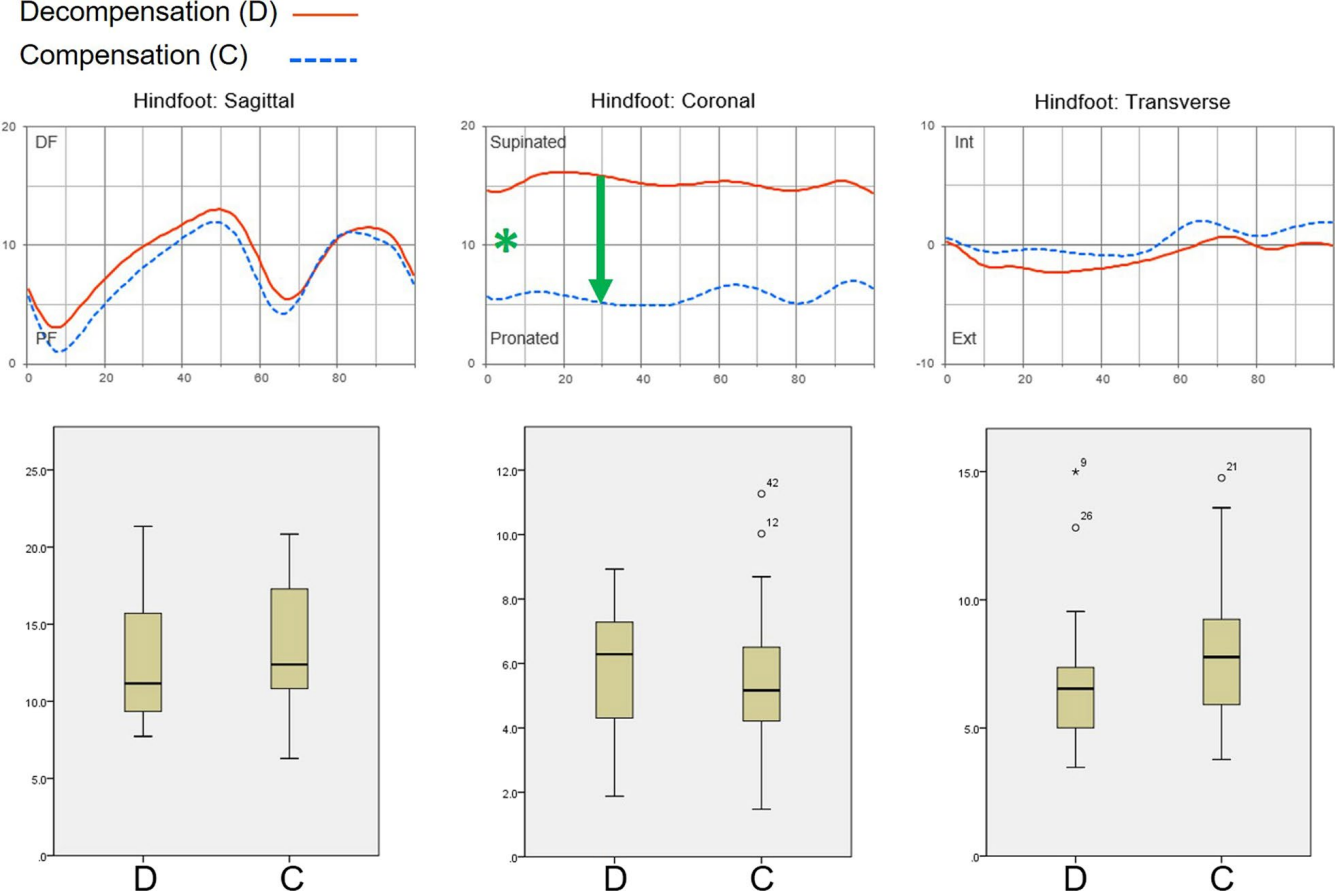
Average hindfoot motion in the subtalar decompensation and compensation groups. There was no intergroup difference in the sagittal and transverse planes. Graphing of the coronal movement of the hindfoot revealed collapsed curves in both groups that differed significantly through all gait phases (star). To differentiate the genuine difference of coronal movement that might be masked by the baseline difference in coronal angle caused by the presence of compensation, we transformed the decompensation curve to a corrected curve by subtracting the difference between the mean angles at the midstance phase of the two curves from all parameters of the decompensation curve. We found no significant difference in any phase between the compensation and corrected decompensation curves

The average motion of the hindfoot (decompensation versus compensation) did not differ significantly in the sagittal and transverse planes throughout all gait phases. There were collapsed curves of coronal hindfoot movement in both groups that revealed a constant significant difference between them throughout all gait phases (Fig. 3). To identify the genuine difference in coronal movement that might be masked by the baseline difference in coronal angle caused by the presence of compensation, we transformed the decompensated curve to a corrected curve by subtracting the difference between the mean angle at the midstance phase of the two curves from all the parameters of the decompensated curve (Fig. 3) [21]. As a result, no significant difference was noted in any of the gait phases between the compensation curve and the corrected decompensation curve.

**Fig. 4:**
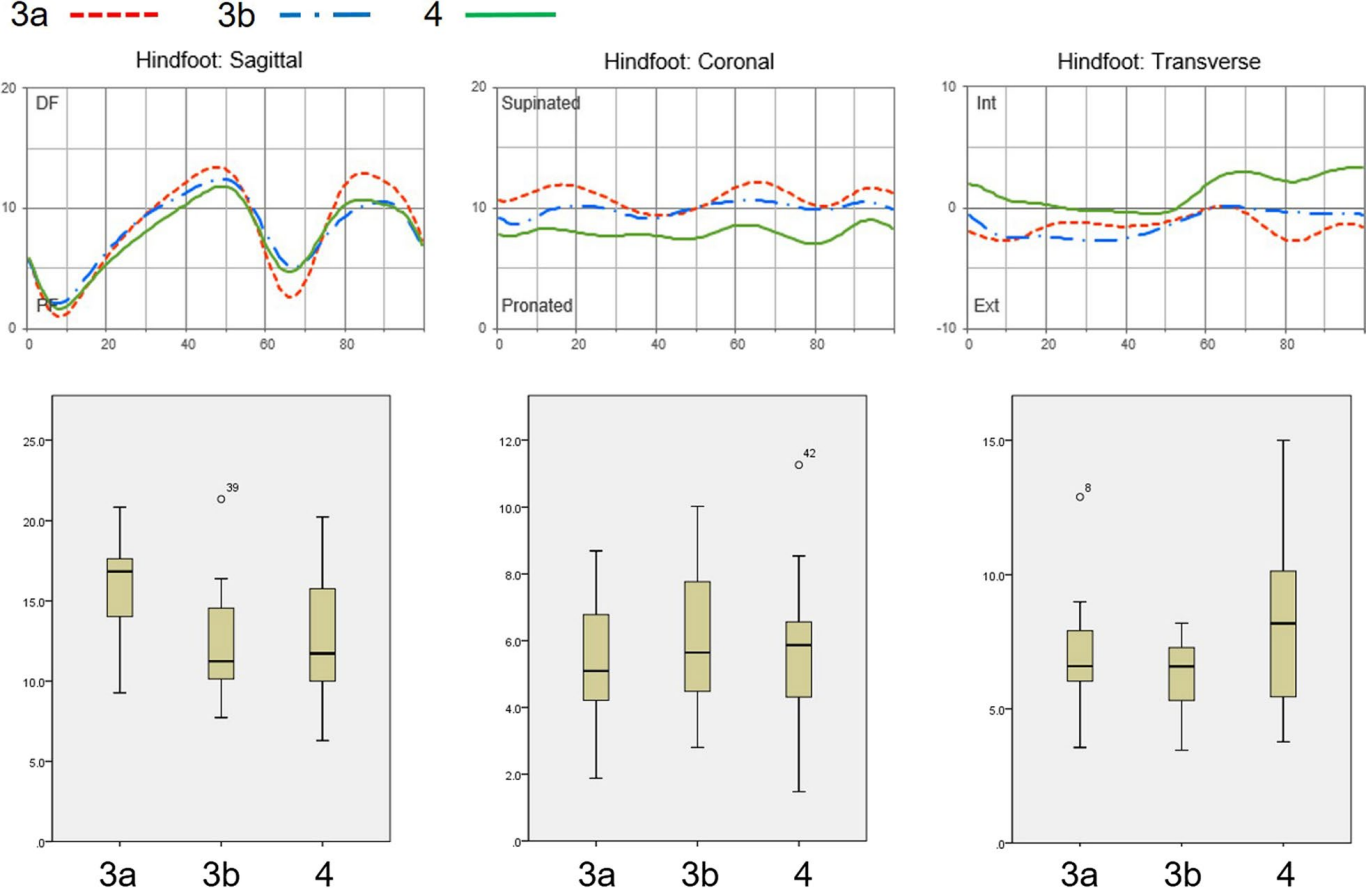
Average hindfoot motion of the three groups (Takakura stage 3a, 3b, and 4). The sagittal range of hindfoot motion was decreased in the cases of more advanced OA (stages 3b, 4) compared with the cases of stage 3a. Otherwise, there were no significant differences between them

Comparison of the three groups by Takakura stage revealed significantly different sagittal ranges of motion among the three groups (Fig. 4; *P* = 0.015). At the more advanced stages of ankle OA (stage 3a to stage 3b), the sagittal range of motion diminished (*P* = 0.028). The mean sagittal range of motion at stage 3b was similar to that at stage 4 (*P* = 1.0). Coronal movement of the hindfoot in Takakura stages 3a/3b/4 showed a relatively flat pattern (Fig. 4). In addition, there were no significant differences among them in all gait phases.

## Discussion

In this study, the hindfoot alignment state determined by subtalar compensation or ankle OA stage did not affect spatiotemporal gait parameters. The sagittal range of hindfoot motion decreased at the advanced ankle OA stage. Coronal hindfoot motion was disrupted through all gait phases regardless of the presence or absence of subtalar compensation. The compensated coronal position of the hindfoot seemed to be maintained throughout the gait cycle.

The spatiotemporal parameters were not affected by the hindfoot alignment resulting from subtalar compensation. Meanwhile, a significant difference in the step width by ankle OA stage was noted in this study. However, this could have been caused by the significant discrepancy in the sex ratio, which could affect the foot and ankle function or structure [22, 23]. We hypothesized that ankle symptoms such as pain degree might not affect their spatiotemporal parameters because all patients included in this study had suffered sufficient long-standing and substantial ankle pain to undergo surgical treatment regardless of ankle OA stage determined on plain radiographs.

Disrupted coronal movement of the subtalar joint had no correlation with ankle OA stage or hindfoot compensation state. Before this study, we expected that the reduced coronal movement of the subtalar joint is naturally led by the reduced sagittal movement of the pathologic ankle joint. However, we still observed limited varus motion at the subtalar joint in patients with a markedly conserved sagittal motion of the ankle joint. We expected that the ankle with neutral hindfoot alignment maintained by subtalar compensation had more residual varus motion reservoirs in several phases such as the preswing phase compared with the ankle with a varus heel led by subtalar decompensation. However, we obtained the opposite result. Once the subtalar motion was disrupted at advanced ankle OA, its biomechanics did not seem to change according to the presence of subtalar compensation or ankle OA stage. Further studies are needed to determine whether surgical treatment, such as total ankle arthroplasty or ankle arthrodesis, could restore the disrupted coronal motion of the subtalar joint in patients with ankle OA.

Decreased sagittal range of motion in stages 3b and 4 compared with stage 3a implied that advanced ankle OA affects the sagittal range of hindfoot motion, although the range of sagittal motion in all three stages seemed to be mostly conserved compared with the ranges of coronal or transverse motion. Takakura stage 3a refers to the obliteration of the medial gutter but the cartilage remaining on the whole talar dome. Stages 3b and 4 commonly have bone contact on the talar dome to greater or lesser degrees. This might have led to the difference in the outcome, although studies with more populations are needed to draw definite conclusions.

We mainly focused on hindfoot rather than midfoot or forefoot motion in this study regarding the analysis using an MFM for two reasons. First, we excluded patients who had a simultaneous pathology of foot joints other than the ankle joint, which was our concern. Second, we believed that the ankle pathology would primarily affect hindfoot motion, while midfoot and forefoot motions would merely show adjustable motion according to abnormal hindfoot movement. To our knowledge, this is the first study to report on hindfoot motion using MFM analysis according to the presence of subtalar compensation and ankle OA stage.

The compensation status of the hindfoot was assessed using a standing simple radiograph in this study. However, utilizing weight-bearing computed tomography (WBCT) allows for a more precise assessment of the alignment of lower limb bones in a standing position [24]. The presence of varus angulation in the knee demonstrated an association with hindfoot valgus, while valgus angulation in the knee exhibited an association with hindfoot varus [25]. Even if the hindfoot appears to be in varus on X-ray, the compensation status of the subtalar joint vary when assessed using WBCT. The calcaneus may exhibit both varus and valgus orientations relative to the talus [19]. Therefore, further research is needed to investigate the relationship between hindfoot alignment assessed by WBCT and MFM analysis in the future.

Our study had several limitations that we could not address. First, because this was a retrospective analysis, we could not evenly assign the number of patients to each group or control sex or laterality in the comparison of the Takakura groups. However, there were no statistically significant differences in the groups sizes. Moreover, the effect of sex or laterality on gait pattern remains debatable. Therefore, we believe that the effects of sex and laterality on this outcome may be limited. Second, we could not separate subtalar motion from whole hindfoot movement in the analysis because we had no available marker set enabling such separation and complete validation. However, we believe that this may not have significantly affected the study outcomes because the coronal movement of the ankle joint is limited compared with that of the subtalar joint. Moreover, ankles with advanced OA are often stiff due to soft-tissue contracture or impingement from the osteophytes of the ankle mortise. Third, we did not include all patients with ankle OA because there were a few cases of early ankle OA in our database, and we excluded several ankles that could not be classified using the Takakura system. Therefore, we might not draw definite conclusions from the study outcomes. Finally, it was limited to assessing compensation solely on the coronal plane, and it did not account the entire lower extremity alignment.

## Conclusion

Spatiotemporal parameters were not affected by the hindfoot alignment resulting from subtalar compensation. The sagittal range of hindfoot motion was decreased in patients with advanced ankle OA. Coronal movement of the subtalar joint was disrupted and was uncorrelated with ankle OA stage or hindfoot compensation state. Further research is needed to investigate the relationship between hindfoot alignment assessed by WBCT and MFM analysis in the future.

## Data Availability

The datasets generated and analyzed during the current study are available from the corresponding author on reasonable request.
